# Fluctuations in direct human presence, not predictable weekly cycles, influence avoidance behaviour in ravens

**DOI:** 10.1186/s40462-026-00671-9

**Published:** 2026-06-03

**Authors:** Varalika Jain, Matthias-Claudio Loretto, Thomas Bugnyar, Petra Sumasgutner

**Affiliations:** 1 Konrad Lorenz Research Center for Behavior and Cognition, Core Facility of the University of Vienna, Grünau im Almtal, 4645 Austria; 2 Department of Behavioral and Cognitive Biology, University of Vienna, Vienna, 1030 Austria; 3 Department of Interdisciplinary Life Sciences, Research Institute of Wildlife Ecology, University of Veterinary Medicine Vienna, Savoyenstraße 1, Vienna, 1160 Austria

**Keywords:** Anthropause, Anthropogenic food sources, Anthropulse, Covid-19-related lockdown, Human disturbance, Foraging, Outdoor recreation, Weekend effect

## Abstract

**Background:**

Rhythms in human activity create patterns of opportunities and disturbances for wildlife, influencing their behaviour and spatial ecology. The weekend effect hypothesis posits that weekday-weekend routines in human activity can impact wildlife in creating cyclic conditions. At our study site – an outdoor wildlife park – free-flying ravens opportunistically exploit food intended for captive animals on a routine basis. While disturbances from visitor activity levels are typically low on weekdays and high on weekends, they can also vary day-to-day. Our access to data detailing visitor numbers makes our context well-suited to test whether ravens anticipate and respond to predictable weekday-weekend patterns following (1) a ‘5 + 2’ structure, or if they respond (2) flexibly to fluctuations in direct human activity levels. By testing the weekend effect hypothesis from the perspective of a ‘5 + 2’ pattern versus flexibly responding to fluctuations in human activity, we better understand wildlife response strategies to human activity.

**Methods:**

Using long-term GPS tracking data, we investigated the weekend effect hypothesis by examining raven foraging probability and space use across two temporal scales: (1) **daily** patterns over five years, encompassing periods of Covid-19 pandemic-related restrictions, and (2) **bihourly** patterns over six weeks. We hypothesized that increased human activity levels would result in lower foraging probabilities and higher space use in ravens, varying between weekdays and weekends.

**Results:**

Our findings provided limited support for the weekend effect hypothesis, considering a ‘5 + 2’ pattern in human activity. However, ravens consistently avoided elevated human activity by decreasing in their probability of foraging at the site and increasing space use on a daily level.

**Conclusions:**

Our study reveals that ravens are behaviourally flexible in their movement responses to disturbance when exploiting anthropogenically created foraging opportunities. Changes influencing anthropogenic resources and disturbances at resources should consider the potential knock-on effects to wildlife.

**Supplementary Information:**

The online version contains supplementary material available at 10.1186/s40462-026-00671-9.

## Background

Rhythms in human activity create spatial and temporal patterns in both opportunities and disturbances for wildlife [1]. These can shape wildlife behaviour and space use, affecting individual animals, entire populations, and whole ecosystems [2–4]. Much like animals adapt to natural environmental cycles, such as circadian or seasonal rhythms [5], they can also respond to human-associated cycles, including daily work schedules and weekly fluctuations, in shared landscapes – as these patterns increase environmental predictability [6, 7]. Opportunities created by human activity rhythms, such as the replenishing of food resources, can attract wildlife to certain areas at certain times [8–11]. Yet, rhythms, such as periodic increases in human presence at resources, often create disturbances that also displace wildlife temporally [12, 13] and spatially [14, 15], particularly when humans are perceived as potential predators [16, 17].

Both opportunity and disturbance cycles can vary in time and space, occurring asynchronously, resulting in nuanced cost-benefit dynamics. Consequently, wildlife must adjust their behaviour in ways that allow them to exploit resources while retaining the ability to avoid or flee perceived danger when necessary [17, 18]. Cognitive mechanisms, such as spatiotemporal memory and learning, can underpin how individuals navigate the dual role of humans as both providers of opportunity and sources of disturbance or threat [18–21]. Such mechanisms vary depending on how different taxa (e.g., ground dwelling versus arboreal versus avian) perceive cues associated with benefit (i.e., food) and cost (i.e., predation risk), giving rise to complex associations and decisions that opportunistic animals have within the human niche [16, 18, 22].

When human activity rhythms follow distinct patterns on weekdays versus weekends, they describe a phenomenon known as the “weekend effect”. The weekend effect hypothesis proposes that animals may learn to anticipate and adapt to these distinct, predictable weekday-weekend patterns [7, 23]. However, studies of the weekend effect differ in their investigation of the hypothesis, complicating our understanding of wildlife responses. For instance, some studies use a traditional Monday-to-Friday weekday and Saturday-Sunday weekend (i.e., ‘5 + 2’ pattern) as a proxy for human activity levels in their analyses [14, 24] – testing for a temporal pattern that may be learnt and remembered by species. However, as ‘5 + 2’ cycles are disrupted by high human activity on public holidays and over vacation periods, others include these additional days as ‘weekends’ into their analyses, testing for behavioural responses to high versus low human activity rather than a temporal pattern [12, 14, 24, 25].

At our study site, a wildlife park (hereafter ‘game park’), humans serve as both an asynchronous source of opportunity and disturbance for a population of free-flying common ravens (*Corvus corax*). These ravens scrounge the food supplied to the captive animals within their enclosures, particularly the wild boars. The park staff leave once they drop off the food, and importantly, this food provisioning occurs independently of the visitors that the game park welcomes all year round. The food opportunity here, following a consistent rhythm of replenishing daily and routinely, can be perceived as a constant, stable resource. The visitors, contrastingly, represent a disturbance. Ravens are generally neophobic and exhibit fear towards unfamiliar humans, likely linked to past or ongoing persecution [26–28]. Like many outdoor recreational areas, the park experiences lower visitor numbers on weekdays and higher visitor numbers on weekends, and thus visitor activity levels tend to follow a ‘5 + 2’ rhythm (Figure S1).

Given the challenges and barriers in collecting and/or accessing data detailing human activity levels, having data on visitor numbers at the park allows us to test how ravens, exploiting a daily opportunity, respond to cycles of disturbance in visitor numbers from two perspectives [29]. First, in whether ravens anticipate and respond to predictable weekday-weekend patterns. Here, we assessed whether raven foraging behaviour and space use differed according to the ‘5 + 2’ temporal cycle of the weekend effect. Second, in whether ravens respond flexibly to high versus low human activity levels. We did so by looking into changes to foraging and movement behaviour in response to fluctuations in visitor numbers.

We used long-term GPS-tracking data to understand how human activity patterns affect an individual’s resource use at two temporal scales – daily and bihourly. We estimated behavioural responses as the ravens’ probability of being at the foraging site (hereafter ‘foraging probability’) and their space use (i.e., capturing potential movements of avoiding or displacing from disturbance). We hypothesised that ravens are negatively affected by human activity levels [30], and would respond to both predictable cycles and fluctuations in human activity by decreasing foraging and increasing space use with higher visitor numbers. On the daily scale, we expected ravens to have stronger negative responses to increasing visitor numbers on weekdays (i.e., typically low numbers), than on weekends (i.e., typically high numbers). As our dataset covered periods of heavily restricted human activity (i.e., reduced disturbance) across the broader landscape from the Covid-19 pandemic lockdowns, we also expected that ravens would differ in their foraging probability and space use with more stringent restriction measures [31]. We expected these restrictions in human activity to affect responses on weekdays and weekends differently, with ravens reacting stronger to human restrictions on weekends (i.e., when human activity is typically high) than on weekdays. On a bihourly scale, we expected a decrease in foraging probability during the peak visitor times of the day, together with weekday-weekend differences.

Our study system reflects many other human-dominated landscapes where wildlife exploits anthropogenic food sources (e.g., garbage dumps and roads), while navigating the challenges of pauses and pulses in human activity levels (e.g., from waste-related activities and traffic) [9, 32, 33]. Testing both a ‘5 + 2’ pattern and direct responses to human activity levels is crucial for understanding the responses of wildlife to asynchronous rhythms of human-associated opportunity and disturbance, managing human-wildlife coexistence, and conserving biodiversity in increasingly human-dominated landscapes.

## Methods

### Study site and population

**Fig. 1:**
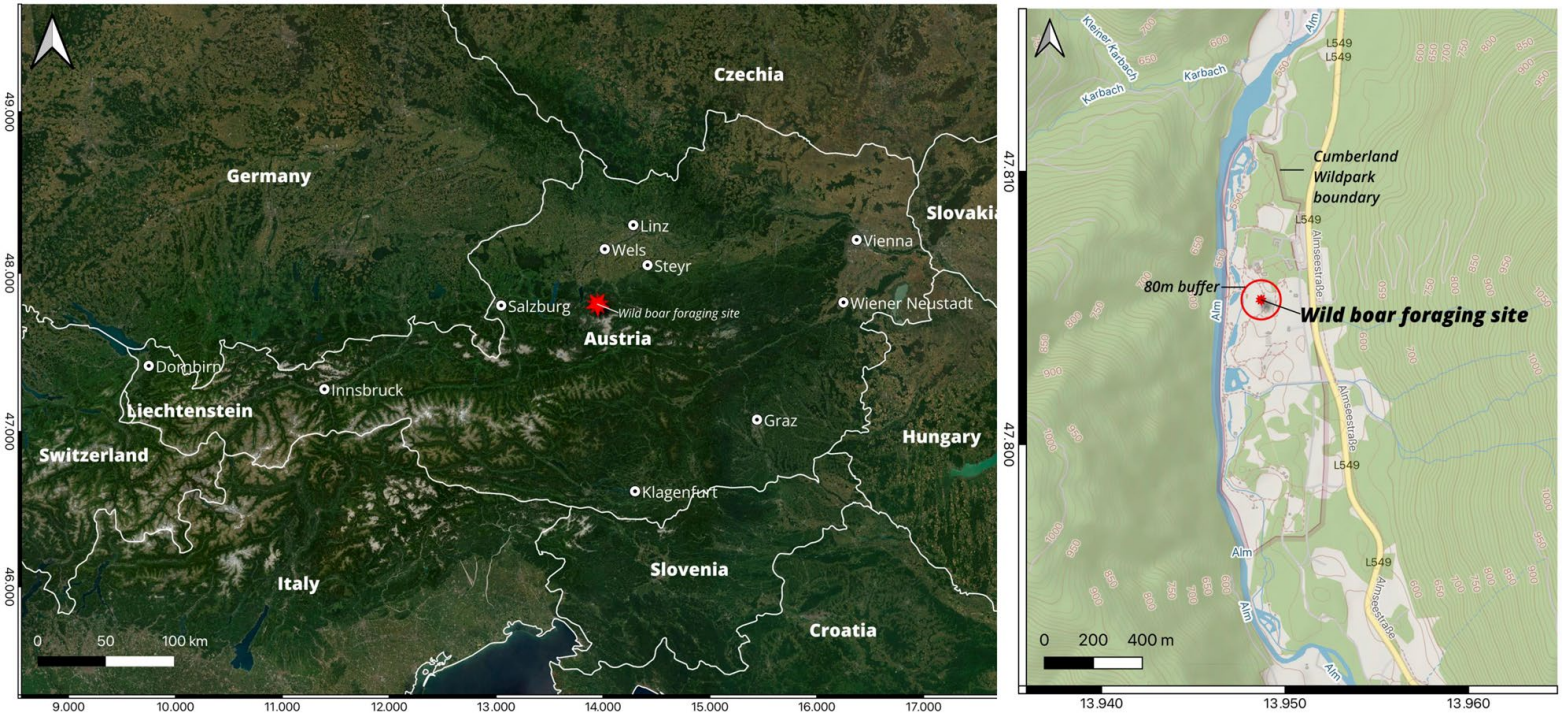
Common ravens (*Corvus corax*) forage daily at a wild boar enclosure (indicated by red star) within Cumberland Wildlife Park, Austria. To understand how human activity impacts the ravens’ foraging behaviour, we constructed an 80 m buffer (red circle) around the wild boar feeding location. Sourcing data from 118 GPS-tagged ravens, we calculated the number of GPS points that intersected with the buffer. We compared the number of fixes in versus out to determine the individual’s probability of being at the foraging site

The Cumberland Wildpark (https://wildpark.at/) is located in the Alm valley, north of the Eastern Alps in Austria, approximately 500 m above sea level (Fig. 1). The outdoor game park covers around 60 hectares and houses European wildlife species and domestic breeds. The park welcomes visitors year-round, from 9am to 5pm from April to October (closing at 7pm), and from 10am to 4pm from November to March (closing at 5pm).

Although ravens frequently scrounge food within various animal enclosures, the wild boar enclosure is visited the most, with ravens attending fixed feeding times twice a day [30]. Ravens, known for their exceptional cognitive and social skills [34–37], repeatedly encounter each other at this foraging site, making it an important hub for social interactions, where social bonds develop and dominance ranks are established [38–40]. The wild boar enclosure provides a low-risk foraging opportunity, with injuries rarely caused by heterospecifics [30, 41, 42]. The boars are fed consistently at one location within the enclosure (47°48’19.08”, 13°56’55.32”) every morning between 8am and 10am, and every afternoon between 3pm and 5pm [41]. Ravens typically gather at the feeding site prior to when feeding occurs, often in the trees within and surrounding the enclosure [43]. The birds rapidly approach the food as soon as the zoo animals are fed (a task completed in approximately 5–10 min) and the staff members depart [41]. Depending on season, approximately 30 ravens are seen in summer and up to 120 in winter [30, 41, 44]. At the park, wild ravens have been caught using drop-in traps baited with meat and bread [45] since the late 90s [39]. Some raven individuals that were initially parent-raised in captivity were also released into free-flight in the same park. These individuals generally integrate successfully into the local non-breeding population within a month or two after release [see methodological details in [46] ].

GPS tagged ravens comprise of sexually immature juveniles (within their first year), sub-adults (2nd and 3rd year), and sexually mature adults (3rd year onwards, mostly non-breeding) [38]. We assigned minimum age estimates based on the coloration of the mouth lining (pink for juveniles, transitioning to black for sub-adults, and black for adults) and plumage (slightly brown for juveniles, black for sub/adults) [47]. Sex was determined genetically from blood samples collected when individuals were trapped.

All study individuals were fitted with backpack-style, solar-powered GPS-transmitters (OrniTrack-25 with elevated solar panels, Ornitela UAB, Lithuania; https://www.ornitela.com/25g-transmitter*)* that weighed 25 g (27 g with Teflon harness and aluminium crimps) and never exceeded 3% of the bird’s bodyweight [38]. Data from the transmitters are downloaded via cell network, stored, and managed on Movebank (https://www.movebank.org/cms/movebank-main) under the study ID: 206,418,248.

### Human activity data

#### Visitor presence

Data on the total number of visitors entering the park each day are routinely collected through ticket sales by the Cumberland Wildpark staff. Data from the beginning of 2018 till the end of 2022 were shared with us for this study (Figures S1a-b).

For 6 weeks from 22nd May 2023 to 2nd July 2023, the park increased the temporal resolution of documenting ticket sales from daily to hourly, providing the opportunity to investigate fluctuations in human activity at a finer temporal resolution. To better align these data to our GPS-data (described further below), we aggregated the hourly visitor presence data into bihourly intervals (e.g., 7 to 9am, 9 to 11am), giving us a measure of bihourly visitor numbers (Figures S1c-d). As we only had a reference for the number of visitors entering the park, and not the duration for which they spent in the park, we estimated two scenarios of visitor presence. In the first, we assumed visitors to spend a short amount of time within the park (1–2 h), staying within the bihourly window at which they entered the park. In the second, we assumed that visitors spent a longer duration at the park (3–4 h), and accounted for this by cumulatively adding the number of visitors entering the park at any given bihourly interval with that of the subsequent bihourly interval.

#### Stringency index

Covid-19 pandemic lockdowns resulted in countrywide changes to human activity [48], and at varying levels of restriction, influenced human activity, particularly in outdoor areas [49, 50]. Previous research has revealed that ravens in this study system exhibit a wide variation in their ranging areas and distances travelled [38, 46, 51]. While we used ticket sales data to capture local-scale changes to human activity at the site, we expected broader-scale fluctuations in human activity to also have an impact on raven behaviour. Especially since periods of high social restriction measures did not always correspond to the park being closed to visitors.

We used the ‘stringency index’ computed by the Oxford Covid-19 Government Response Tracker (OxCGRT) to capture these broader-scale lockdown-related changes to human activity [52]. The stringency index is a composite index capturing the stringency of lockdown restrictions measures, such as travel bans, scaling from 0 (no restrictions on human activity) to 100 (strictest restrictions on human activity). We assigned a stringency index value of 0 to the dates prior to the pandemic.

### GPS data

We sourced 5 years of GPS data, from the 1st of January 2018 to the 31st of December 2022, to match the daily ticket sales data, and 6 weeks of data, from the 22nd of May 2023 to the 30th of July 2023, to match the data on bihourly visitor numbers.

The GPS transmitters were programmed to collect data every 15-minutes, however during periods of limited solar recharge (e.g., in winter), the sampling rate reduced to every 1–2 h, and during periods of very low battery, to even longer sampling intervals. In 2018 and 2019, as part of a pilot study, the transmitters were programmed to collect data at a much higher sampling rate (ranging from every 1 s to 10 min; Figure S2). We therefore down-sampled all the data to ensure regular intervals at a minimum of 15 min. We also filtered the data to only daytime data, defined by sunrise and sunset start [53].

Autocorrelation arises when sequential GPS-fixes along a trajectory show similar characteristics, particularly when close together in time [54, 55]. To test for autocorrelation in our dataset, we first isolated individuals with a consistent 15-minute (± 2 min) sampling interval on any given day (*n* = 8,890). For these individuals, we tested for autocorrelation in three movement characteristics: step length (i.e., distance between successive GPS fixes or steps), turning angle (i.e., the relative angle between two successive steps), and speed (between two successive steps) [54]. We found that most of the data exhibited a decrease in autocorrelation at a lag of 30-minutes [also see [55] ] (Figures S3 and S4). We thinned our data to a minimum 30-minute interval.

As we wanted to understand the effects of human activity on ravens at a specific foraging site, we had to define a meaningful distance threshold at which we would [1] not capture absences from individuals who didn’t visit the site due to being spatially far away (i.e., would give us a false impression of avoidances), yet [2] retain those that were spatially close enough to visit the site but didn’t (i.e., absences reflecting potential disturbance-avoidance behaviour). To achieve this, we first calculated the maximum distance an individual had from the wild boar feeding site on any given day. Of the individuals who visited the site at least once in a day, we found that 99.9% of their maximum distances were concentrated within a 50 km radius that encompasses known anthropogenic food sources [see [46] for further details of these known food sources] (Figure S5). We therefore used the 50 km radius as our distance threshold, whereby individuals whose entire daily trajectories were more than 50 km away from the site were considered to be too spatially far away and removed from the dataset. Given that ravens are social foragers who move fast between known food resources, where potential information exchange can occur, we considered any individual whose daily trajectory was in part or entirely within the 50 km radius to be possibly influenced by human activity changes at the site [38, 56].

#### Foraging probability

Following Jain et al. (2022) [46], we defined an 80 m buffer around the wild boar feeding location, beyond which individuals were considered not to be using the foraging site (Figure S6). We then measured the number of fixes inside versus outside the buffer to define our first response variable – the probability of an individual being at the resource (i.e., foraging probability). We calculated the foraging probability daily and for each bihourly interval within a day. Due to sampling irregularities, we only considered daily foraging probabilities from individuals that had at least 6 GPS-fixes per day – a cut-off that on the shortest day in winter reflected a GPS-point per hour from sunrise to sunset. From the bihourly intervals, as we had 1–2 GPS-fixes per hour at a minimum of 30-minute sampling interval, we only used data that had 4 out of 4 GPS-fixes to estimate bihourly foraging probabilities.

To capture the daily space use of individuals, we used their trajectories to calculate the area encompassed by the 95% Kernel Density Estimate (KDE) [i.e., 95% of their location probability distribution [57] ]. As KDEs are sensitive to sample sizes [58], we defined the KDEs for individuals with at least 15 fixes in a day. We determined this threshold of 15 fixes by conducting a down-sampling and KDE-calculation exercise on 5% of the individuals in our dataset (*n* = 88 out of 1,676 individual-date combinations) that had the highest number of GPS-fixes in a day (*n* = 32). We randomly down-sampled these daily trajectories 20 times to various sample sizes (6 to 30 at intervals of 2) and computed the KDEs for each sample size and repeat. We assessed the robustness of the resulting KDEs by comparing its estimated area and proportion of overlap to the ‘full’ KDE (i.e., considering all 32 points within that day; Figure S7). We expected the relationship between the area / proportion overlap and the reduced sample size to stabilise at a point, reflecting a piecewise linear relationship. Using a segmented or broken-line regression, we determined the minimum sample size at which KDE estimates would stabilise and be reliable, resulting in 15 GPS fixes per day, with some variation (area: mean = 12 fixes, range: 8–26; proportion overlap: mean = 12 fixes, range: 8–26; Figure S8) [59].

We conducted all GPS data processing in R (version 4.3.3 [60]). The ‘suncalc’ package was used to filter daytime datapoints [53]. Autocorrelation was determined using the ‘acf’ function [54]. The 80 m foraging buffer was determined using the ‘recurse’ package [61]. Foraging probabilities were estimated using the ‘move’ [62], ‘sp’ [63] and ‘sf’ [64] packages, and space use was calculated using the ‘amt’ package [65]. The segmented regression was conducted using the ‘segmented’ package [59].

### Statistical analysis

We ran a set of generalised linear mixed models (GLMM, Table 1) to understand how ravens’ **foraging probability** (model 1 [m1]) and **space use** (model 2 [m2]) varied in response to human activity on a **daily level**, and a generalised additive mixed model (GAMM) to understand how **foraging probability** varied on a **bihourly level** when visitors stayed for a short period in the park (model 3a [m3a]) versus a longer period (model 3b [m3b]). We used a GAMM for the bihourly model to allow for non-linearity to be accounted for in the temporal bihourly predictor [66, 67].

**Table 1:** Descriptions of the model structures to understand how common ravens (*Corvus corax*) alter their daily foraging probability (m1) and space use (m2), and bihourly foraging probability (m3) in response to human activity. Human activity was specified as three test fixed effects in the model (in bold) – type of day, ticket sales / visitors at the park, and the stringency index

Response variable	Rationale		Daily models Generalised linear mixed model		Bihourly model Generalised additive mixed model	
Type of day (weekday, weekend)	Test for weekend versus weekday differences in human activity	Response variable	Foraging probability (m1)	Space use (m2)	Foraging probability assuming short visitor duration (m3a)	Foraging probability assuming long visitor duration (m3b)
Ticket sales / visitors (covariate)	Test for responses to fluctuations in human activity (local scale)	Distribution	Beta-family (logit link)	Gaussian	Binomial (logit link)	Binomial (logit link)
Stringency index (covariate)	Test for responses to human activity during lockdowns (broad scale)	Full model	**type of day** x **ticket sales** + **type of day** x **stringency** + origin x **ticket sales** + origin x **stringency** + sex + age class + day of year		**type of day** x **visitors** + origin x **visitors** + sex + age class + bihourly	
Origin (captive bred, wild-caught)	Control for differences based on rearing environment	Random effects	(1 + type of day x ticket sales + type of day x stringency | ID) + (1 + Origin + sex + age class | date)		(1 + type of day x visitors | ID) + (1 + Origin + sex + age class | date)	
Age class: (juvenile, sub-adult, adult)	Control for age-related differences	Null model	origin + sex + age class + day of year		origin + sex + age class + bihourly	
Sex: (male, female)	Control for sex-related differences	Reduced model	**type of day** + **ticket sales** + **stringency** + origin + sex + age class + day of year	**type of day** x **stringency** + origin x **stringency** + sex + age class + day of year		
Day of year / bihourly interval	Control for temporal changes					

#### Daily foraging probability and space use

On the **daily level**, we tested for the fixed effects of type of day (factor in two levels: weekday, weekend), ticket sales (covariate) and stringency index (covariate), and the interactions between type of day and the latter two covariates on **foraging probability** (m1) and **space use** (m2). We scaled and centred the covariates – ticket sales and stringency index – for easier interpretability [68] and to ease model convergence. In both models, we controlled for the interaction between origin (factor in two levels: captive-released, wild-caught) and ticket sales, and origin and stringency index. We suspected that captive-released birds might respond to changes in human activity differently to wild-caught birds. We also controlled for the fixed effects of sex (factor in two levels: male, female) and age class (factor in three levels: sub-adult and adult; juveniles were classified to sub-adults in March), to account for cohort-differences linked to dominance and experience [40]. We accounted for seasonal variation by turning the day of year into radians (dividing by 365.25 and multiplying with 2π), and including the sine and cosine of the resulting variable into the model at 1x (one peak and trough) and 2x (two peaks and troughs) frequencies [69].

We included the random intercepts of the individual identity and date in both models. All theoretically identifiable random slopes in the model were included (Table 1) to avoid overconfident model estimates and to keep type I error rates at the nominal level of 5% [70, 71]. Specifically, in both **daily foraging probability** (m1) and **space use** (m2) models, we included the random slopes of type of day, ticket sales, and stringency index, and their interactions, and season within individual. We included origin, sex, and age class as random slopes within date. The **foraging probability** data comprised of 35,828 observations from 130 individual ravens with approximately 5,118 observations per estimated fixed effect. The **space use** data comprised of 30,781 observations from 123 individual ravens with approximately 4,397 observations per estimated fixed effect.

We modelled the daily **foraging probability** (m1) using a beta family distribution with logit link function [72–74]. We weighted the model by the total number of fixes daily (i.e., we gave more weight to a response supported by more GPS fix locations). Prior to fitting the daily foraging probability model, we transformed the response (y) by the sample size (n) using the formula ($\:y\bullet\:\left(n-1\right)+0.5)/n$ to narrow the data between, but excluding, zero and one – to fit the beta distribution [75, 76]. We modelled daily **space use** (m2) using a Gaussian distribution (i.e., linear model), also weighted by the total number of fixes daily [75, 76]. Prior to fitting the daily space use model, we log-transformed the response to achieve a symmetrical distribution.

#### Bihourly foraging probability

On the **bihourly level**, we tested for the fixed effects of type of day, visitor numbers (covariate), and their interaction on **foraging probability** for visitors entering the park for a short duration (m3a) and a longer duration (m3b). We scaled and centred the covariate, visitor numbers. As with the previous models, we controlled for the interaction between origin and visitor numbers, and the fixed effects of sex and age class. Non-linear temporal changes were accounted for by including the bihourly interval of the day (covariate from 1 to 6 corresponding to: ‘7–9’, ‘9–11’, ‘11–13’, ‘13–15’, ‘15–17’, and ‘17–19’ hours) in the model using a smooth function represented by a basis size of 6 [66, 67].

We included the random intercepts of the individual identity and date in the model. We included the random slopes of type of day, visitor number, and their interaction within individual. We included visitor number, origin, sex, and age class as random slopes within date. In both visitor duration scenarios, the **bihourly data** comprised of 4,118 observations from 22 individuals across 6 weeks, with 275 observations per estimated fixed effect.

As we only considered data from intervals with the maximum number of GPS-fixes recorded (*n* = 4), we modelled **bihourly foraging probability** for short (m3a) and long (m3b) visitor duration using a binomial distribution (logit link), where the response (points inside versus outside) was captured using the ‘cbind’ function.

#### Modelling approach

We began with fitting maximal models for all the responses, where we included the parameters for the correlations among random slopes and intercepts [70]. However, both the **daily** and **bihourly foraging probability** models (m1, m3a, m3b) failed to converge when all correlations were considered. We therefore excluded correlations among random slopes and intercepts from the foraging probability models [77]. For the **daily space use** model (m2), we used the maximal model.

We fitted the daily models in R (version 4.3.3 [60]), using the ‘glmmTMB’ function from ‘glmmTMB’ [78], and the bihourly models using the ‘gamm4’ function from ‘gamm4’ [66]. For the gamm4 models, we called on the ‘mer’ object for model evaluation, which describes the fitted model object returned by ‘glmer’ [66]. We did not find any indication for overdispersion, assessed by a dispersion parameter < 1.2, for non-Gaussian distributions, and by visual inspection of Q-Q plots (of residuals against fitted values) for Gaussian distributions [79]. All models contained more than one predictor and so we tested for collinearity issues by specifying a model without interaction terms or smooths and by using Variance Inflation Factors (VIF) that were consistently < 2 [80, 81].

As an overall test of the fixed effects of interest we conducted a full-null model comparison aimed at avoiding cryptic multiple testing (Table 1) [82]. In the **daily models** (m1, m2), the null model comprised of only origin, sex, age class, and day of year. In the **bihourly models** (m3a, m3b), the null model comprised of only origin, sex, age class, and bihourly interval. The null models had the same random effect and slope structure as the full model. The comparison was based on a likelihood ratio test [83] and only if this revealed significance, we further investigated the influence of individual predictor variables. Additionally, for models satisfying the full-null model comparison, we removed non-significant interaction terms to be able to interpret the respective lower order terms included within it. Along with the model output, we report the significance of individual fixed effects by means of likelihood ratio tests [83], comparing the full model with models lacking the terms of interest one at a time (R function ‘drop1’). Lastly, we report the confidence intervals of model estimates and fitted values.

## Results

We analysed the **foraging probability** (m1) of 130 ravens and the **space use** (m2) of 123 ravens across 5 years on a **daily level.** Ravens exhibited great variation in their daily foraging probabilities (average: 0.18, range: 0.00–1.00) and daily space use (average: 120.38 km^2^, range: 490.93 m^2^ – 53,447.02 km^2^). Very large probabilistic space use estimates appeared for individuals who travelled long distances within the day and should not be interpreted within the context of long-term range sizes (Figure S9). We also analysed the bihourly foraging probability for 22 individuals across 6 weeks under two scenarios of visitor duration at the park – short (m3a) and long (m3b). Ravens also exhibited variation in their bihourly foraging probabilities (average: 0.14, range: 0.00–0.86).

We found that the full-null model comparisons for the daily foraging probability (m1: χ^2^ = 16.77, df = 7, *p* = 0.019) and space use (m2: χ^2^ = 36.50, df = 7, p = < 0.001) were significant, indicating that the test predictors meaningfully contributed to explaining the variability in the response variables. However, the full-null model comparisons for the bihourly foraging probabilities were insignificant for both short (m3a: χ^2^ = 5.023, df = 4, *p* = 0.285) and long (m3b: χ^2^ = 4.397, df = 4, *p* = 0.355) visitor duration. We therefore did not further investigate the influence of individual predictor variables for the bihourly foraging probabilities.

### Daily foraging probability

In the daily foraging probability model (m1), we did not find significance in any of the interactions: type of day and ticket sales (χ^2^ = 1.695, df = 1, *p* = 0.193), type of day and stringency index (χ^2^ = 0.219, df = 1, *p* = 0.640), origin and ticket sales (χ^2^ = 0.830, df = 1, *p* = 0.362), and origin and stringency index (χ^2^ = 3.312, df = 1, *p* = 0.069) (Tables S1-S2).

**Fig. 2:**
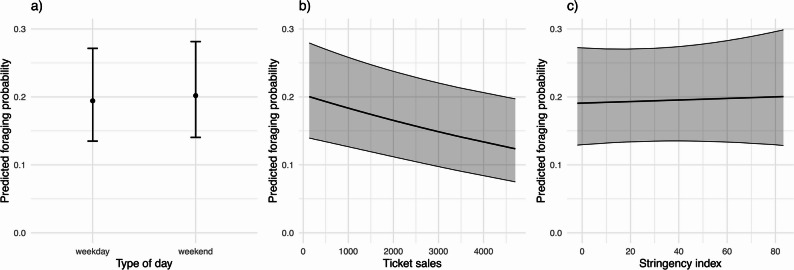
The predicted daily foraging probabilities of common ravens at the wild boar feeding site (**a**) did not differ between weekdays and weekends; (**b**) decreased with increasing ticket sales; (**c**) did not change with increasing stringency index

After removal of the non-significant interaction terms, to interpret the effects of lower order terms (Table S3), we found that the type of day and ticket sales influenced foraging probabilities. While type of day (χ^2^ = 4.162, df = 1, *p* = 0.041) did meaningfully explain variation in the response, the difference between weekends and weekdays was marginal. We interpret the output as lacking a clear ‘5 + 2’ pattern (Fig. 2a). Foraging probability, however, did show a trend of decreasing with increasing ticket sales (i.e. higher human activity) (χ^2^ = 9.187, df = 1, *p* = 0.002) (Fig. 2b). The stringency index did not influence foraging probabilities (χ^2^ = 0.071, df = 1, *p* = 0.790) (Fig. 2c).

Amongst the control predictors, we found origin (χ^2^ = 10.013, df = 1, *p* = 0.002) and age (χ^2^ = 12.215, df = 2, *p* = 0.002) differences in foraging probabilities, but no significant variation between sexes (χ^2^ = 1.188, df = 1, *p* = 0.276). Wild-caught birds had a lower foraging probability than captive-released birds, and sub-adults had the highest foraging probability, followed by juveniles and adults (Fig. 2e). Lastly, we observed strong seasonality (χ^2^ = 892.772, df = 4, p = < 0.001) in the foraging probabilities, peaking around spring and early summer months and dipping in autumn.

### Daily space use

In the daily space use model (m2), we did not find significance in the interactions between type of day and ticket sales (χ^2^ = 1.395, df = 1, *p* = 0.238), nor between origin and ticket sales (χ^2^ = 0.040, df = 1, *p* = 0.842) (Tables S4-S5).

**Fig. 3:**
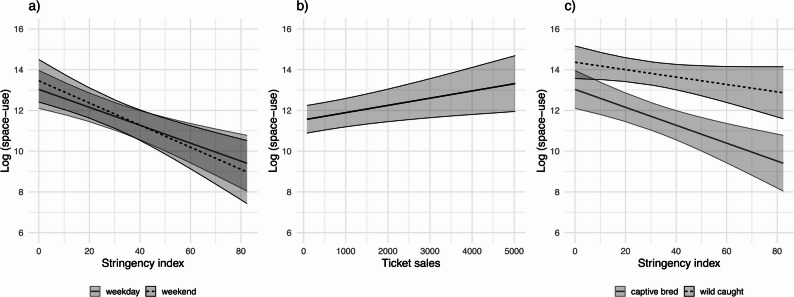
The predicted daily space use of common ravens at the wild boar feeding site (**a**) decreased at a slightly steeper rate on weekends than weekdays with increasing stringency index values; (**b**) increased with increasing ticket sales; (**c**) was higher for wild-caught birds than captive-released individuals, and decreased at a steeper rate for captive-released than wild-caught birds

After removal of the non-significant interaction terms (Table S6), we found that space use decreased along a slightly steeper slope on weekends than weekdays with increasing stringency index (χ^2^ = 7.837, df = 1, *p* = 0.005). We interpret this output as lacking a clear ‘5 + 2’ pattern (Fig. 3a). We also observed space use increasing with greater ticket sales (χ^2^ = 7.300, df = 1, *p* = 0.007) (Fig. 3b). Wild-caught birds had a higher space use than captive-released individuals, and the space use of captive-released individuals decreased more steeply than wild caught individuals with increasing stringency index values (χ^2^ = 4.132, df = 1, *p* = 0.042) (Fig. 3c).

We also found sex (χ^2^ = 5.300, df = 1, *p* = 0.021) and age (χ^2^ = 96.152, df = 2, p = < 0.001) differences in space use. Males had a greater space use than females, and juveniles had a smaller space use compared to sub-adults and adults (please note that at a minimum sample size of 15 GPS-fixes per day, data from juveniles were relatively lower in the dataset, particularly in winter months). There was also seasonality (χ^2^ = 741.787, df = 4, p = < 0.001) in space use, dipping in summer and peaking in autumn.

## Discussion

In this study, we investigated how ravens, while exploiting a consistent human-provided food source, responded to rhythms of human disturbance at an outdoor recreational site. Our findings indicate that ravens respond more to fluctuations in visitor numbers occurring at the site than to a ‘5 + 2’ weekday-weekend pattern. We observed that ravens decreased in their daily probability of foraging at the site and increased their daily space use with increasing visitor numbers. Responses to daily human activity fluctuations, as opposed to a temporal rhythm, suggest that ravens adjust their behaviour to avoid or displace from disturbance as it becomes prominent, as opposed to adjusting their behaviour in anticipation of patterned disturbance cycles [18].

Behavioural flexibility is a prerequisite for wildlife to access human-modified landscapes, especially when novel opportunities and threats arise in asynchrony [84]. The lack of a clear weekday-weekend pattern in the ravens’ daily foraging probabilities and space use – despite their pronounced responses to direct changes human activity – suggests that ravens exhibit behavioural flexibility and mediate disturbances by displacing from areas of high human activity. This aligns with previous work showing flexibility in ravens across multiple domains, including cognitive [85], foraging [43], social [40], movement [51], and ecological flexibility [8]. Ravens are also highly attracted to anthropogenic food sources, as illustrated by their returning to the resource following deliberate disturbances (e.g., non-lethal shots) [86]. In our system, this capacity for flexibility may be particularly advantageous when exploiting a highly attractive and predictable resource [7, 38, 86]. In other words, when rewards are consistent and of quality, foraging at those resources can be a time/ energy-saving strategy, and therefore the ability to adjust behaviour in light of disturbances likely pays off [87]. Other corvid species have been shown to flexibly modify foraging behaviour at anthropogenic resources, balancing risk and reward rather than abandoning profitable sites altogether by minimising exposure to disturbance while maximising energetic gain per unit time [88–90]. Moreover, because visitor numbers do not consistently map onto simple weekday-weekend patterns, successful exploitation of resources may require ravens to respond adaptively to human presence [15, 21].

Employing a flexible response strategy may also be advantageous when animals can access a broader network of resources that help mitigate the costs of local disturbances [38, 91, 92]. From the trajectories of ravens who visited the park on any given day, we observed that they also appear to travel to alternative anthropogenic food sources, up to roughly 50 km away and sometimes even further. This is consistent with previous research demonstrating that ravens can visit multiple resources within a single day [38, 46]. Ravens in our study, despite responding negatively to high human activity, may therefore buffer local disturbances by exploiting alternative resources in the surrounding landscape, such as garbage dumps and compost sites [44, 46]. Studies on other avian scavenging species exploiting anthropogenic food sources have demonstrated that individuals can flexibly reallocate foraging effort across a network of spatially dispersed natural and anthropogenic resources, allowing them to withstand localized disturbances or resource loss [33, 93, 94].

Interestingly, although ravens travelled widely within a day, their behaviour appeared to be more strongly shaped by local human activity at the foraging site than by the broad-scale changes in human activity associated with Covid-19 pandemic restrictions [3, 95]. The stringency index showed no effect on overall foraging probability. However, captive-bred released individuals did reduce their space use during periods of higher restriction. Resource exploitation and responses to disturbance can vary depending on individual experience, dominance rank, and life-history stage, making certain groups within a population respond differently to human activity than others [96]. While both captive-bred and wild-caught ravens subsist on human resources across the landscape [44, 46], captive-bred individuals – raised by their parents and released into free flight (next to the study site) as juveniles at dispersal – use the study site more intensively than wild caught conspecifics. In some species, such as black-kites, *Milvus migrans*, exposure to humans is associated with heightened aggression towards humans around food rewards [97]. However, even though captive-bred birds in our study population were experienced with the presence of humans from their upbringing, they hardly had any direct contact with humans inside the aviaries and, already as recently fledged young, showed clear avoidance responses to approaching persons in flight initiation tests (unpublished observations). Their reduction in space use during high restriction periods may therefore reflect heightened site fidelity given unfamiliar changes in the broader landscape and reduced disturbance at the foraging site. In contrast, wild-caught individuals were consistent in their space use at varying levels of restriction, perhaps due to their established knowledge on alternative anthropogenic resources across the landscape [92], which may have remained unaffected by pandemic restrictions.

In response to disturbances, species have been observed to shift their activity at resources to quieter periods to temporally avoid periods of human disturbance [13, 24, 90]. We detected temporal patterns at the daily scale, but not at the finer, bihourly scale. Given the asynchrony between food provisioning and disturbance within a day (i.e., ravens scrounge food from the wild boars before the park opens up to visitors), we expected foraging probabilities to be highest in the mornings, and then to vary over the course of the day, declining as visitor numbers peaked. However, our uncertainty about how long visitors remained in the park, combined with the relatively coarse temporal resolution of our GPS data at the bihourly scale, likely limited our ability to capture meaningful within-day responses.

## Conclusions

Our investigation of the weekend effect hypothesis, in relation to both the type of day and human activity levels, underscores that ravens do not anticipate weekday-weekend patterns, but rather that ravens respond flexibly to high versus low human activity levels. Our study demonstrates that responding flexibly may be a preferred strategy for species exploiting beneficial anthropogenic resources associated with disturbances, and this can slightly differ within or across different groups (captive-bred versus wild) of a population. These conclusions open up further questions regarding the cognitive mechanisms driving behavioural flexibility at anthropogenic food sites, including whether ravels rely solely on immediate environmental cues when balancing foraging opportunities against disturbance risk. Ravens are renowned for their excellent social memory in experimental settings, differentiating between specific humans after months and even years [98, 99]; they are also renowned for their planning skills in respect to challenges faced at foraging [92, 100, 101]. Yet, hardly anything is known about how they use their cognitive abilities in coping with human activity on a large scale in daily life. Future studies could investigate whether ravens apply episodic-like memory, future planning, or delay-of-gratification strategies when exploiting anthropogenic food resources [102–104]. Such questions also relate to broader themes in cognitive movement ecology, including how cognition shapes habitat selection, movement decisions, and responses to predictable human disturbance [105]. Finally, management changes influencing either the human patterns of resource provisioning or the occurrence of disturbances should consider the knock-on effects to wildlife sharing these resources.

## Supplementary Information

Below is the link to the electronic supplementary material.

Supplementary Material 1

## Data Availability

The GPS datasets downloaded for this study are visible in the Movebank Data Repository (study ID: 206418248). The processed datasets used for statistical analyses and the corresponding code are available on Zenodo at [DOI: 10.5281/zenodo.18365465, Github link to code https://github.com/vee-jain/Raven_weekend.git].

## Abbreviations

GLMM: Generalised linear mixed model
KDE: Kernel Density Estimate
OxCGRT: Oxford Covid-19 Government Response Tracker
