# Supplementary material for: Fluctuations in direct human presence, not predictable weekly cycles, influence avoidance behaviour in ravens
Source: Mov Ecol. 2026 Jun 3;14:56. doi: 10.1186/s40462-026-00671-9 (PMC13450542; doi:10.1186/s40462-026-00671-9)
Supplement: Supplementary file 1 [file 40462_2026_671_MOESM1_ESM.docx]

Fluctuations in direct human presence, not predictable weekly cycles, influence avoidance behaviour in ravens

**Supplementary information**

**Figure S1** (a-b) Daily ticket sales data from the wildlife park shows that visitor numbers tend to be higher on the weekends than weekdays. On a bihourly scale, the number of visitors at the park is also higher on weekends than weekdays, and peaks in the morning when assuming (c) short 1-2 hour-long visits to the park, and peaks in middle of the day when assuming (d) long 3-4 hour-long visits to the park.

**Figure S2** The average daily sampling interval from all individuals in the dataset, with the red dashed horizontal line indicating an interval of 15-minutes. In 2018 and 2019, data were collected at more frequent intervals as part of a pilot study. Note that the y-axis is limited to 60-minutes to better visualise the data – longer sampling intervals persist in the dataset due to periods of low transmitter battery.

**Figure S3** a) Step length, b) turning angle, and c) speed [autocorrelation](https://www.sciencedirect.com/topics/agricultural-and-biological-sciences/autocorrelation) plot for one individual raven (Alfon on 2019-05-18) with a consistent 15-minute sampling interval throughout the day.

**Figure S4** Mean and error of [autocorrelation](https://www.sciencedirect.com/topics/agricultural-and-biological-sciences/autocorrelation) in a) step-length, b) turning angle, and c) speed, for all individuals that had a consistent 15-minute sampling interval on any given day (n = 8,890).

**Figure S5** Daily trajectories of ravens (in blue) who visited the study site (pink marker) at least once in a day. Of the individuals who visited the site at least once in a day, 99.9% of their maximum distances were concentrated within a 50km radius (red circle) that encompasses known anthropogenic food sources (white and black square markers).

**’**

**Figure S6** Following Jain et al. (2022), we used the ‘recurse’ package to examine how the number of revisitations at each identified AFS changed with buffer sizes up to 150 m in radius, beyond which individuals were not considered to be using the site.

**Figure S7** We randomly down-sampled the trajectory of Rollo from 2021-06-20 (n=32) to a range of sample sizes (6 to 30 at intervals of 2), repeated 20 times. For each iteration of random down-sampling at each reduced sample size, we computed the 95% Kernel Density Estimate (KDE). We assessed the robustness of the resulting KDEs by comparing its (a) estimated area and (b) proportion of overlap to the ‘full’ KDE considering all 32 points within that day. (c) The KDEs resulting from the down-sampling at multiple reduced sample sizes (columns) is shown for 5 repeats (rows).

**Figure S8** Example of the broken-line regression (red line) for (a) estimated area and (b) proportion overlap for the randomly down-sampled trajectory of Rollo from 2021-06-20 (n=32). The modelled break point is represented by a vertical dashed line.

**Figure S9** 95% daily Kernel Density Estimates (KDEs) were large for raven individuals who travelled far distances within a day while also visiting the foraging site in that day. The blue line was fitted using the ‘stat_smooth’ function from ggplot2 to illustrate the relationship between KDE and the maximum displacement away from the site.

**Table S1** Results of the full model addressing the effects of human activity patterns at a recreational site - type of day (i.e., weekday versus weekend cycles), ticket sales (i.e., human activity levels), and the stringency index (i.e., restriction on human activity) – on common ravens’ daily foraging probability. Estimates are presented together with standard errors, 95% confidence intervals, and significance tests.

| **Term** | **Model output** | | | | | **Likelihood ratio test** | | |
| --- | --- | --- | --- | --- | --- | --- | --- | --- |
|  | **Estimate** | **SE** | **lower CI** | **upper CI** | **p** | **Chisq** | **df** | **p** |
| Intercept | -1.810 | 0.222 | -2.245 | -1.375 | <0.001 |  |  |  |
| Type of day^†^ | 0.050 | 0.024 | 0.004 | 0.097 | 0.033 |  |  |  |
| Ticket sales^‡^ | -0.015 | 0.025 | -0.064 | 0.033 | 0.541 |  |  |  |
| Origin^*^ | -0.822 | 0.243 | -1.299 | -0.345 | 0.001 |  |  |  |
| Stringency index^‡^ | 0.184 | 0.119 | -0.049 | 0.416 | 0.122 |  |  |  |
| Sex^∆^ | 0.244 | 0.241 | -0.227 | 0.716 | 0.310 | 1.028 | 1 | 0.311 |
| Age (juvenile)^§^ | -0.002 | 0.050 | -0.099 | 0.096 | 0.974 | 12.286 | 2 | 0.002 |
| Age (sub-adult) | 0.078 | 0.027 | 0.026 | 0.130 | 0.003 |  |  |  |
| Sin (day of year) | 0.317 | 0.015 | 0.287 | 0.347 | <0.001 | 891.241 | 4 | <0.001 |
| Cos (day of year) | -0.319 | 0.023 | -0.364 | -0.275 | <0.001 |  |  |  |
| Sin (2 x day of year) | -0.098 | 0.015 | -0.128 | -0.069 | <0.001 |  |  |  |
| Cos (2 x day of year) | 0.054 | 0.017 | 0.021 | 0.086 | 0.001 |  |  |  |
| Type of day^†^: ticket sales^‡^ | -0.030 | 0.023 | -0.076 | 0.015 | 0.194 | 1.695 | 1 | 0.193 |
| Origin^*^: ticket sales^‡^ | -0.022 | 0.025 | -0.071 | 0.026 | 0.369 | 0.830 | 1 | 0.362 |
| Type of day^†^: stringency index ^‡^ | 0.010 | 0.021 | -0.032 | 0.052 | 0.640 | 0.219 | 1 | 0.640 |
| Origin^*^: stringency index^‡^ | -0.289 | 0.154 | -0.592 | 0.013 | 0.061 | 3.312 | 1 | 0.069 |

*‡ = z-transformed to a mean of zero and a standard deviation of one; mean and sd of the original ticket sales were 360.45 and 433.93, and of the stringency index were 29.30 and 28.08 respectively*

*† = type of day with ‘weekday’ as the reference category*

** = origin with ‘captive-released’ as the reference category*

*∆ = sex with ‘female’ as the reference category*

*§ = age class with ‘adult’ as the reference category*

**Table S2** Results of the full model on common ravens’ daily foraging probability with regard to the random effects (estimated standard deviation).

| **Grouping variable** | **Effect^*^** | **sd** |
| --- | --- | --- |
| Individual identity | Intercept | 1.274 |
|  | Type of day | 0.038 |
|  | Ticket sales | 0.075 |
|  | Stringency index | 0.578 |
|  | Type of day: ticket sales | 0.055 |
|  | Type of day: stringency index | 0.000 |
| Date | Intercept | 0.215 |
|  | Origin | 0.152 |
|  | Sex | 0.314 |
|  | Age (juvenile) | 0.434 |
|  | Age (sub-adult) | 0.175 |

** 'intercept' depicts to a random intercept, all others to a random slope*

**Table S3** Results of the reduced model on common ravens’ daily foraging probability, lacking the interactions between type of day and ticket sales, type of day and stringency index, origin and ticket sales, and origin and stringency index. The numbers depicted in bold show significant results.

| **Term** | **Model output** | | | | | **Likelihood ratio test** | | |
| --- | --- | --- | --- | --- | --- | --- | --- | --- |
|  | **Estimate** | **SE** | **lower CI** | **upper CI** | **p** | **Chisq** | **df** | **p** |
| Intercept | -1.825 | 0.222 | -2.260 | -1.391 | 0.000 |  |  |  |
| Type of day^†^ | 0.048 | 0.023 | 0.002 | 0.094 | 0.039 | **4.162** | **1** | **0.041** |
| Ticket sales^‡^ | -0.045 | 0.015 | -0.074 | -0.016 | 0.002 | **9.187** | **1** | **0.002** |
| Stringency index^‡^ | 0.021 | 0.081 | -0.137 | 0.180 | 0.791 | 0.071 | 1 | 0.790 |
| Origin* | -0.789 | 0.243 | -1.264 | -0.313 | 0.001 | **10.013** | **1** | **0.002** |
| Sex^∆^ | 0.262 | 0.240 | -0.208 | 0.733 | 0.275 | 1.188 | 1 | 0.276 |
| Age (juvenile)^§^ | 0.000 | 0.050 | -0.097 | 0.098 | 0.994 | **12.215** | **2** | **0.002** |
| Age (sub-adult) | 0.079 | 0.027 | 0.026 | 0.131 | 0.003 |  |  |  |
| Sin (day of year)^ø^ | 0.316 | 0.015 | 0.286 | 0.346 | 0.000 | **892.772** | **4** | **<0.001** |
| Cos (day of year) | -0.324 | 0.022 | -0.368 | -0.280 | 0.000 |  |  |  |
| Sin (2 x day of year) | -0.096 | 0.015 | -0.125 | -0.067 | 0.000 |  |  |  |
| Cos (2 x day of year) | 0.054 | 0.017 | 0.021 | 0.087 | 0.001 |  |  |  |

*‡ = z-transformed to a mean of zero and a standard deviation of one; mean and sd of the original ticket sales were 360.45 and 433.93, and of the stringency index were 29.30 and 28.08 respectively*

*† = type of day with ‘weekday’ as the reference category*

** = origin with ‘captive-released’ as the reference category*

*∆ = sex with ‘female’ as the reference category*

*§ = age class with ‘adult’ as the reference category*

**Table S4** Results of the full model addressing the effects of human activity patterns at a recreational site - type of day (i.e., weekday versus weekend cycles), ticket sales (i.e., human activity levels), and the stringency index (i.e., restriction on human activity) – on common ravens’ daily space use. Estimates are presented together with standard errors, 95% confidence intervals, and significance tests.

| **Term** | **Model output** | | | | | **Likelihood ratio test** | | |
| --- | --- | --- | --- | --- | --- | --- | --- | --- |
|  | **Estimate** | **SE** | **lower CI** | **upper CI** | **p** | **Chisq** | **df** | **p** |
| Intercept | 13.086 | 0.345 | 12.411 | 13.761 | 0.000 |  |  |  |
| Type of day^†^ | 0.146 | 0.106 | -0.063 | 0.355 | 0.170 |  |  |  |
| Ticket sales^‡^ | 0.275 | 0.132 | 0.017 | 0.534 | 0.037 |  |  |  |
| Origin^*^ | 2.114 | 0.379 | 1.371 | 2.857 | 0.000 |  |  |  |
| Stringency index^‡^ | -1.275 | 0.325 | -1.912 | -0.638 | 0.000 |  |  |  |
| Sex^∆^ | 0.843 | 0.362 | 0.133 | 1.553 | 0.020 | 5.339 | 1 | 0.021 |
| Age (juvenile)^§^ | -0.843 | 0.098 | -1.035 | -0.650 | 0.000 | 95.872 | 2 | <0.001 |
| Age (sub-adult) | 0.122 | 0.048 | 0.028 | 0.216 | 0.011 |  |  |  |
| Sin (day of year) | -0.383 | 0.033 | -0.448 | -0.317 | 0.000 | 743.125 | 4 | <0.001 |
| Cos (day of year) | 0.790 | 0.046 | 0.700 | 0.879 | 0.000 |  |  |  |
| Sin (2 x day of year) | 0.266 | 0.035 | 0.198 | 0.335 | 0.000 |  |  |  |
| Cos (2 x day of year) | -0.504 | 0.039 | -0.580 | -0.428 | 0.000 |  |  |  |
| Type of day^†^: ticket sales^‡^ | -0.191 | 0.162 | -0.507 | 0.126 | 0.238 | 1.395 | 1 | 0.238 |
| Origin^*^: ticket sales^‡^ | -0.013 | 0.065 | -0.140 | 0.114 | 0.841 | 0.040 | 1 | 0.842 |
| Type of day^†^: stringency index ^‡^ | -0.268 | 0.115 | -0.493 | -0.043 | 0.020 | 6.022 | 1 | 0.014 |
| Origin^*^: stringency index^‡^ | 0.724 | 0.369 | 0.001 | 1.447 | 0.050 | 3.905 | 1 | 0.048 |

*‡ = z-transformed to a mean of zero and a standard deviation of one; mean and sd of the original ticket sales were 365.99 and 407.03, and of the stringency index were 30.41 and 28.36 respectively*

*† = type of day with ‘weekday’ as the reference category*

** = origin with ‘captive-released’ as the reference category*

*∆ = sex with ‘female’ as the reference category*

*§ = age class with ‘adult’ as the reference category*

**Table S5** Results of the full model on common ravens’ daily space use with regard to the random effects (estimated standard deviation).

| **Grouping variable** | **Effect^*^** | **sd** |
| --- | --- | --- |
| Individual identity | Intercept | 1.845 |
|  | Type of day | 0.812 |
|  | Ticket sales | 1.193 |
|  | Stringency index | 2.278 |
|  | Type of day: ticket sales | 1.539 |
|  | Type of day: stringency index | 0.829 |
| Date | Intercept | 1.632 |
|  | Origin | 1.282 |
|  | Sex | 1.081 |
|  | Age (juvenile) | 2.148 |
|  | Age (sub-adult) | 1.716 |
| Residual |  | 1.509 |

** 'intercept' depicts to a random intercept, all others to a random slope*

**Table S6** Results of the reduced model on common ravens’ daily space use, lacking the interactions between type of day and ticket sales, and origin and ticket sales. The numbers depicted in bold show significant results.

| **Term** | **Model output** | | | | | **Likelihood ratio test** | | |
| --- | --- | --- | --- | --- | --- | --- | --- | --- |
|  | **Estimate** | **SE** | **lower CI** | **upper CI** | **p** | **Chisq** | **df** | **p** |
| Intercept | 13.039 | 0.343 | 12.367 | 13.711 | 0.000 |  |  |  |
| Type of day^†^ | 0.106 | 0.100 | -0.089 | 0.302 | 0.287 |  |  |  |
| Ticket sales^‡^ | 0.130 | 0.047 | 0.037 | 0.222 | 0.006 | **7.300** | **1** | **0.007** |
| Stringency index^‡^ | -1.247 | 0.322 | -1.877 | -0.617 | 0.000 |  |  |  |
| Origin^*^ | 2.117 | 0.378 | 1.377 | 2.857 | 0.000 |  |  |  |
| Sex^∆^ | 0.838 | 0.362 | 0.128 | 1.548 | 0.021 | **5.300** | **1** | **0.021** |
| Age (juvenile)^§^ | -0.844 | 0.098 | -1.037 | -0.651 | 0.000 | **96.152** | **2** | **<0.001** |
| Age (sub-adult) | 0.122 | 0.048 | 0.028 | 0.216 | 0.011 |  |  |  |
| Sin (day of year)^ø^ | -0.384 | 0.033 | -0.449 | -0.319 | 0.000 | **741.787** | **4** | **<0.001** |
| Cos (day of year) | 0.786 | 0.046 | 0.697 | 0.875 | 0.000 |  |  |  |
| Sin (2 x day of year) | 0.269 | 0.035 | 0.200 | 0.337 | 0.000 |  |  |  |
| Cos (2 x day of year) | -0.504 | 0.039 | -0.580 | -0.427 | 0.000 |  |  |  |
| Type of day^†^: stringency index ^‡^ | -0.294 | 0.113 | -0.515 | -0.073 | 0.009 | **7.837** | **1** | **0.005** |
| Origin^*^: stringency index^‡^ | 0.732 | 0.365 | 0.017 | 1.448 | 0.045 | **4.132** | **1** | **0.042** |

*‡ = z-transformed to a mean of zero and a standard deviation of one; mean and sd of the original ticket sales were 365.99 and 407.03, and of the stringency index were 30.41 and 28.36 respectively*

*† = type of day with ‘weekday’ as the reference category*

** = origin with ‘captive-released’ as the reference category*

*∆ = sex with ‘female’ as the reference category*

*§ = age class with ‘adult’ as the reference category*
